# Successful Endovascular Aneurysm Repair Using a Balloon-Expandable Stent Graft Bridging the Endograft Limb and a Small External Iliac Artery

**DOI:** 10.70352/scrj.cr.25-0605

**Published:** 2026-06-26

**Authors:** Yusuke Fujioka, Koichi Morisaki, Kentaro Inoue, Daichi Ito, Mitsuru Nakanishi, Kohei Ueno, Go Kinoshita, Shinichiro Yoshino, Tomoharu Yoshizumi

**Affiliations:** Department of Surgery and Science, Graduate School of Medical Sciences, Kyushu University, Fukuoka, Fukuoka, Japan

**Keywords:** aneurysm, endovascular aneurysm repair, stent graft, access trouble

## Abstract

**INTRODUCTION:**

Oversizing of a stent graft limb is one of the risk factors for limb graft occlusion after endovascular aneurysm repair (EVAR) for abdominal aortic and iliac artery aneurysms. We report a case of common iliac artery (CIA) aneurysm with a shrunken external iliac artery (EIA) treated with a Gore C3 Excluder (W. L. Gore & Associates, Flagstaff, AZ, USA) and VBX (W. L. Gore & Associates).

**CASE PRESENTATION:**

A 76-year-old man was incidentally diagnosed with a right CIA aneurysm (diameter = 55 mm) on CT examination. He had a history of spinal cord injury at the age of 33 years, which paralyzed the lower part of his body. Owing to disuse atrophy, his right EIA became smaller with a diameter of 5 mm. The patient was treated with a Gore Excluder C3, and to prevent oversizing of the distal landing zone, a 6-mm VBX was used as the right stent graft limb. The postoperative course was uneventful, and 1 year after EVAR, the stent graft limb was patent.

**CONCLUSIONS:**

This case report describes successful endovascular treatment using Excluder C3 and VBX for CIA aneurysm with a shrunken EIA. In the case of a small-caliber EIA, the use of VBX may help avoid oversizing of the stent graft limb, which may prevent stent graft limb occlusion.

## Abbreviations

ABI
: ankle–brachial index
CIA
: common iliac artery
EIA
: external iliac artery
EVAR
: endovascular aneurysm repair

## INTRODUCTION

EVAR is a widespread, less invasive treatment for abdominal aortic and iliac artery aneurysms.^1–3)^ However, a stent graft has anatomical restrictions and is not suitable for all cases. One of the major challenges for conventional EVAR is hostile proximal neck anatomy or iliac access. Among many causes that include iliac artery angulation, tortuosity, calcification, and stent material, oversized stent graft is one of the risk factors for stent graft limb occlusion.^4,5)^ The smallest commercially available stent graft limb is 10 mm in diameter, which is comparable with a native artery diameter of 8–9 mm. Therefore, an EIA smaller than 7 mm is not recommended for use with commercially available stent grafts. In this report, we present a case of EVAR for CIA aneurysm with a shrinking EIA due to paraplegia caused by spinal cord injury.

## CASE PRESENTATION

Right CIA aneurysm was detected incidentally in a 76-year-old man by CT examination. He had a history of paralysis of the lower part of the body owing to a spinal cord injury at the age of 33 years. ABI measurement was attempted, but a reliable value could not be obtained. CT imaging revealed a spindle-shaped right CIA aneurysm with a diameter of 55 mm. Due to disuse atrophy, the right EIA exhibited a small diameter of 5 mm (**Fig. 1A**–**1D**). The ipsilateral internal iliac artery was patent (**Fig. 1E**). Considering the non-ambulatory status of the patient, EVAR was selected as the treatment. Our strategy was to use GORE VIABAHN VBX (W. L. Gore & Associates, Flagstaff, AZ, USA) as a landing on the shrunken right EIA.

**Fig. 1:**
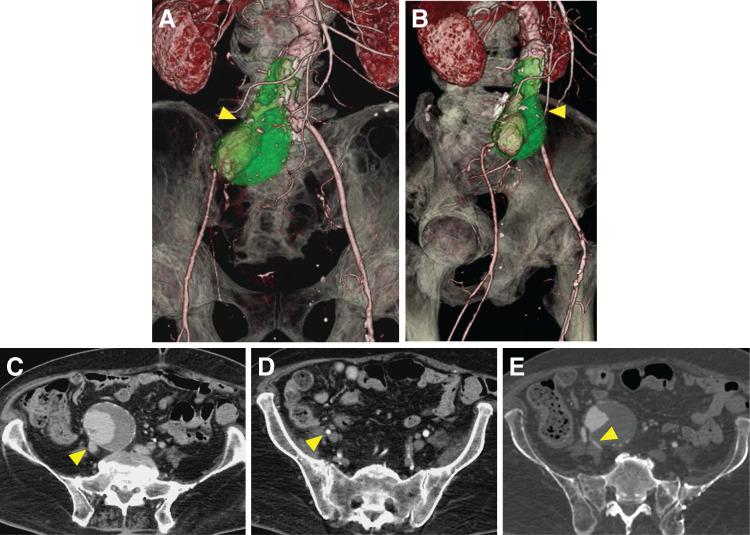
Preoperative CT images. (**A** and **B**) Preoperative CT images showing a spindle-shaped right CIA aneurysm with a diameter of 55 mm. The arrowhead indicates the spindle-shaped right CIA aneurysm. (**C** and **D**) There is a caliber difference between the CIA and the EIA, with the right EIA being 5 mm in diameter. The arrowhead in panel (**C**) indicates the CIA, and the arrowhead in panel (**D**) indicates the EIA. (**E**) The right internal iliac artery was patent. CIA, common iliac artery; EIA, external iliac artery

EVAR was performed percutaneously under general anesthesia. First, a Gore C3 Excluder (W. L. Gore & Associates) with a main body of 26 mm–12 mm–12 cm was inserted and deployed via the left common femoral artery. After contralateral gate cannulation, a GORE VIABAHN VBX, 6–79 mm, was deployed from the right EIA to the CIA (**Fig. 2A**). Then, a contralateral leg 12 mm–14 cm was deployed from the contralateral leg of the main body to the proximal part of the VBX. The aneurysmal change extended to the bifurcation of the CIA; therefore, extending the peripheral sealing zone of the main device to the EIA was mandatory to achieve an adequate distal seal. Ballooning of the overlapping region between the contralateral leg and the VBX was performed using a Mustang 8 mm × 4 cm balloon (Boston Scientific, Marlborough, MA, USA) (**Fig. 2B** and **2C**). The overlap length between the contralateral leg and the VBX was 44 mm. The final expanded inner diameter was 8 mm for the overlapping zone with the Excluder leg and 6 mm for the EIA landing zone. Finally, the ipsilateral limb of the main body was fully deployed to the EIA, and ballooning was performed using the the MOB balloon (W. L. Gore & Associates). Angiography showed no endoleaks (**Fig. 2D**).

**Fig. 2:**
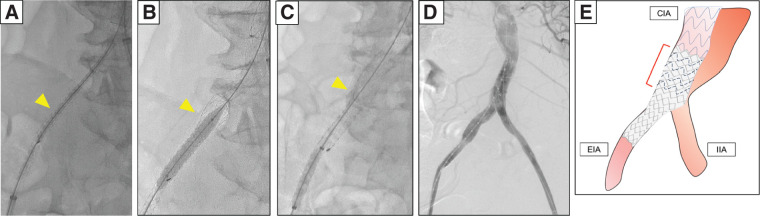
Intraoperative images. (**A**) A GORE VIABAHN VBX 6–79 mm was deployed from the right EIA to the CIA. The yellow arrowhead indicates the deployed device. (**B** and **C**) Ballooning of the overlapping region between the contralateral leg and the VBX was performed. The yellow arrowhead indicates the overlapping region. (**D**) Angiography showed no endoleaks. (**E**) Schema of stent graft placement. The red line indicates the overlapping region. CIA, common iliac artery; EIA, external iliac artery; IIA, internal iliac artery

Postoperatively, ABI improved to 0.95. CT scan on POD 5 revealed no endoleaks, including type III, and good blood flow in the bilateral lower extremities (**Fig. 3A**–**3C**). The postoperative course was uneventful, and a year after EVAR, the stent graft limb was patent.

**Fig. 3:**
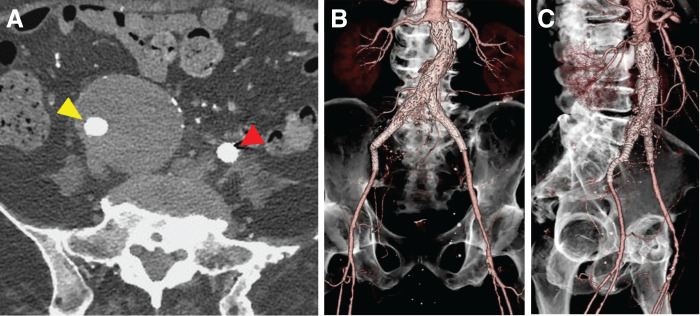
Postoperative CT images showing (**A**–**C**) no endoleaks and reasonable blood flow in the bilateral lower extremities. (**A**) The yellow arrowhead indicates the right EIA, and the red arrowhead indicates the left EIA. EIA, external iliac artery

## DISCUSSION

In this study, we report a case in which CIA aneurysm with a small EIA access was treated using Excluder C3 and VBX.

Stent graft oversized by more than 15% of the native vessel size is one of the risk factors for stent graft limb occlusion, along with iliac artery angulation, tortuosity, calcification or stenosis, small abdominal aortic aneurysm or narrow aortic bifurcation, and stent graft material.^6–11)^ Therefore, avoiding the use of an oversized stent graft is important to prevent limb graft occlusion. Pre-balloon dilatation and/or stenting is a useful technique for EIA stenosis caused by atherosclerosis^12)^; however, these techniques are not applicable for fundamentally small vessels because of the risk of dissection and vessel injury. The minimum diameter of a commercially available stent graft limb is 10 mm, and that of the target vessel is 8–9 mm. If the patient in this case had been treated with a commercial stent graft limb, there would have been 100% oversizing because the diameter of the right EIA was shrunk to 5 mm. Consequently, we used VBX, a commercially available balloon-expandable stent graft, as the EIA landing. VBX has a unique plasticity and can attain a funnel shape with partial post-ballooning. This feature enabled the VBX to have 2 sizes simultaneously: 8 mm for the overlapping zone with the Excluder leg, and 6 mm for the part of the EIA landing. The procedure we followed is useful for avoiding oversizing.

Reports describing endovascular treatment for severely atrophic or hypoplastic iliac arteries remain limited. Orrico et al. reported EVAR in a patient with poliomyelitis-associated unilateral lower-limb atrophy and a markedly hypoplastic iliac artery.^13)^ In that case, a branched iliac device was deployed at the infrarenal aortic level, and 2 VBX balloon-expandable stent grafts were used to bridge the side branch of the device to the internal iliac artery, thereby preserving pelvic perfusion in the setting of severely reduced iliac vessel caliber. Akai et al. also described successful EVAR in a patient with an extremely narrow iliac access route.^14)^ Because a standard contralateral limb could not reach the contralateral gate, 2 VBX stent grafts were sequentially deployed from the CIA and extended proximally to the main body, functioning as a contralateral limb component.

In these limited reports, VBX has been used for branch reconstruction or as a limb-forming device in patients with atrophic or hypoplastic iliac arteries. In contrast, in the present case, VBX was deployed continuously from the CIA into the EIA, overlapping with the endograft limb proximally and extending distally into the small EIA to establish a distal landing zone. This configuration allowed a gradual diameter transition from the endograft limb to the small EIA, thereby avoiding oversizing.

In addition, in the present case, preoperative ABI measurement was attempted; however, a reliable value could not be obtained. The patient exhibited impaired lower-limb perfusion, as indicated by a decreased ABI. Therefore, the intervention was not performed solely for EVAR but also served as treatment for peripheral artery disease, which further supported the use of VBX in this case.

Before VBX became available, the only way to prevent stent graft limb occlusion due to oversizing was to reinforce the limb with a bare metal stent.^15)^ Certainly, this method is a useful procedure for preventing stent graft limb occlusion in cases of oversizing. However, in this procedure, both a stent graft limb and a bare metal stent are required because of the treatment length, which makes it more expensive than a single VBX. Thus, from an economic point of view, treatment with VBX was chosen.

## CONCLUSIONS

This case report describes a successful endovascular treatment using Excluder C3 and VBX for CIA aneurysm with a shrunken EIA. In the case of a small-caliber EIA, the use of VBX can help avoid oversizing of the stent graft limb, which may help prevent stent graft limb occlusion.
